# A Large Secondary Umbilical Endometriosis (Villar's Nodule): A Case Report

**DOI:** 10.1002/ccr3.73397

**Published:** 2026-08-22

**Authors:** Babak Mamaghani, Mohsen Hasanzadeh, Amirmohammad Khodaei, Zeinab Shakibaee Fard

**Affiliations:** ^1^ Clinical Research Development Center Imam Reza Hospital, Kermanshah University of Medical Sciences Kermanshah Iran; ^2^ Student Research Committee, Medical School Kermanshah University of Medical Sciences Kermanshah Iran

**Keywords:** cesarean section, cyclical umbilical pain, estrogen‐dependent lesion, extrapelvic endometriosis, umbilical endometriosis, umbilical mass, Villar's nodule

## Abstract

Umbilical endometriosis is a rare extrapelvic manifestation of endometriosis, presenting diagnostic and surgical challenges. We report a case of a 43‐year‐old woman with a history of two cesarean sections who presented with a progressively enlarging, painful umbilical mass associated with cyclical symptoms and bloody discharge. The primary objective of this report is to highlight the diagnostic pitfalls and surgical decision‐making in large umbilical endometriosis. Preoperative evaluation suggested an irreducible umbilical hernia. Surgical exploration revealed a large lesion extending to the fascia, which was completely excised and subsequently confirmed as umbilical endometriosis on histopathology. This case underscores the importance of including umbilical endometriosis in the differential diagnosis of umbilical masses in women of reproductive age.

## Key Clinical Message

Umbilical endometriosis should be considered in women presenting with cyclical umbilical pain, swelling, or bleeding, especially after prior abdominal surgery. Clinical examination may underestimate lesion extent because deeper fascial involvement can remain occult. Complete surgical excision with histopathological confirmation remains the gold standard for diagnosis and treatment.

## 1 Introduction

Umbilical endometriosis (UE), also known as Villar's nodule, was first described by Villar in 1886 and represents a rare form of extrapelvic endometriosis characterized by the presence of functional endometrial glands and stroma within the umbilical region. UE is classified as either primary, occurring in the absence of previous abdominal or pelvic surgery, or secondary, developing after surgical procedures such as cesarean section, laparoscopy, or laparotomy as a result of iatrogenic implantation of endometrial tissue. Although secondary UE may present several years after the inciting surgery, delayed presentation has been well documented in the literature [1, 2].

Secondary umbilical endometriosis is an uncommon manifestation of extragenital endometriosis and is most frequently associated with prior abdominal surgery, particularly cesarean section, supporting the theory of iatrogenic implantation of endometrial tissue. Although it represents a small proportion of extragenital endometriosis cases, it remains clinically important due to its diagnostic difficulty and potential for misdiagnosis as other umbilical conditions [3]. Recent case reports continue to highlight these diagnostic challenges, especially when the lesion mimics more common surgical entities such as umbilical hernia [4].

Clinically, UE most commonly presents as an umbilical mass associated with cyclical pain, swelling, and occasionally bleeding or discharge that correlates with the menstrual cycle. However, due to its rarity and nonspecific presentation, diagnosis is often delayed or missed, and patients may initially be evaluated for more common conditions affecting the umbilicus [2].

Preoperative imaging, particularly ultrasonography, may assist in assessing lesion size, depth, and relationship with surrounding structures; however, it is not definitive for diagnosis. Therefore, histopathological examination following surgical excision remains the gold standard for confirmation of UE [5, 6]. Fine‐needle aspiration cytology has limited diagnostic value and may yield inconclusive results in a significant proportion of cases [2].

Management is primarily surgical, with complete excision recommended to achieve definitive diagnosis, symptom relief, and reduction of recurrence risk. Medical therapy may provide temporary symptom control but is generally not curative [7, 8]. Given the potential association with pelvic endometriosis in a subset of patients, further evaluation may be considered in selected cases based on clinical suspicion.

In this report, we present a rare case of large secondary umbilical endometriosis initially misdiagnosed as an irreducible umbilical hernia, highlighting the diagnostic pitfalls, surgical considerations, and importance of considering UE in the differential diagnosis of umbilical masses.

## 2 Case History/Examination

A 43‐year‐old woman presented with a one‐year history of intermittent periumbilical pain that worsened cyclically with menstruation. Recently, she experienced a marked increase in pain intensity along with the onset of bloody discharge from the umbilicus, accompanied by nausea and vomiting. Her medical history was notable for two previous cesarean sections performed several years earlier. On physical examination, a firm, immobile subcutaneous mass measuring approximately 4 × 4 cm was palpated beneath the umbilicus, with overlying brownish, edematous skin containing two sinus‐like openings (Figure 1). Tenderness was localized to the umbilical region, with no abnormalities noted elsewhere in the abdomen.

**FIGURE 1:**
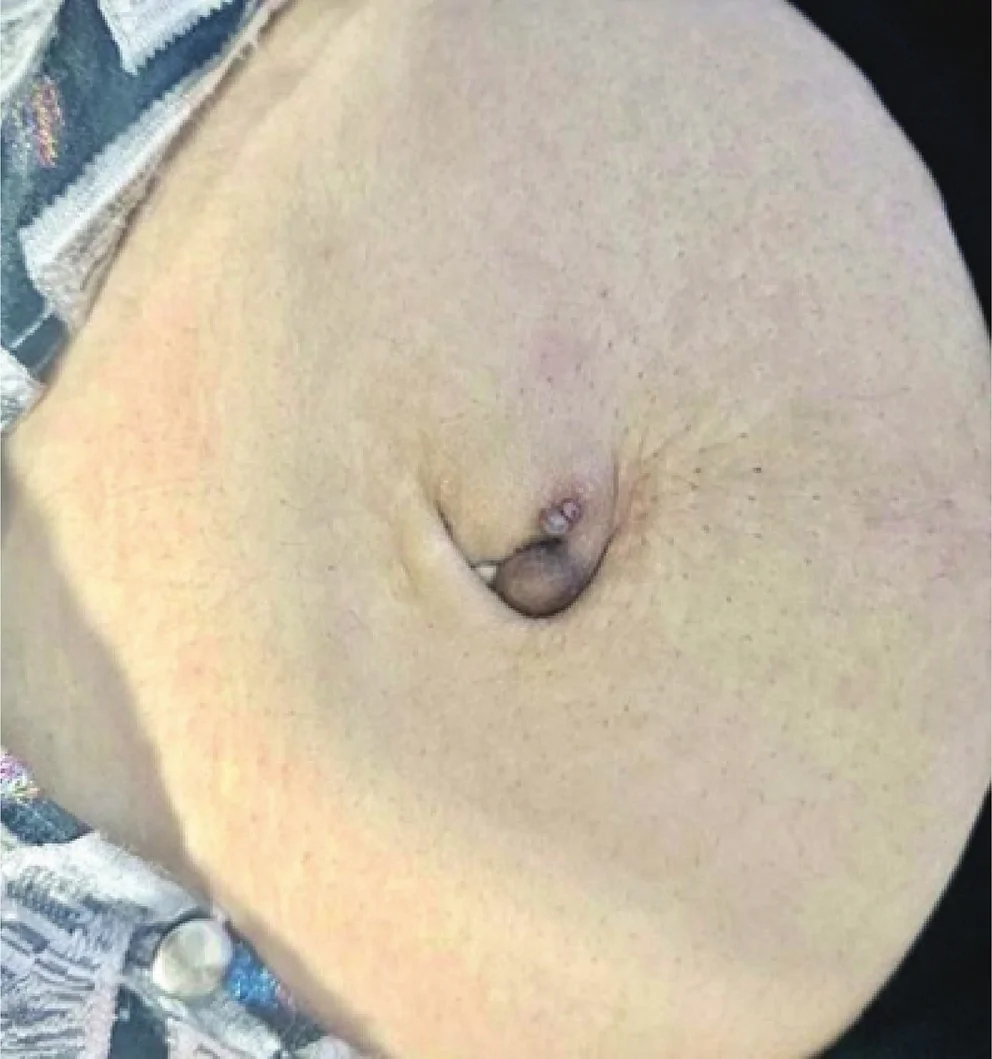
Gross appearance of the umbilical lesion. A firm, brownish nodular mass measuring approximately 4 × 4 cm is visible beneath the umbilicus, with overlying edematous skin and two sinus‐like openings. Patient consent was obtained for photography.

## 3 Differential Diagnosis, Investigation and Treatment

Abdominal ultrasonography showed an umbilical hernia containing mesenteric fat but no bowel loops, with a fascial defect measuring 20 mm. The lesion remained nonreducible despite gentle probe pressure. Dynamic assessment during the Valsalva maneuver demonstrated no reduction of the protruding tissue. Based on these findings, a provisional diagnosis of an irreducible umbilical hernia was made, prompting surgical exploration.

During surgery, an elliptical incision around the umbilicus allowed exposure of a firm, fibrotic, brownish mass measuring approximately 6.5 × 5 × 3.1 cm, adherent to the anterior rectus sheath (Figure 2). The discrepancy between the clinical and intraoperative measurements can be explained by the deeper extension of the lesion into the fascia, which was not fully appreciable on physical examination.

**FIGURE 2:**
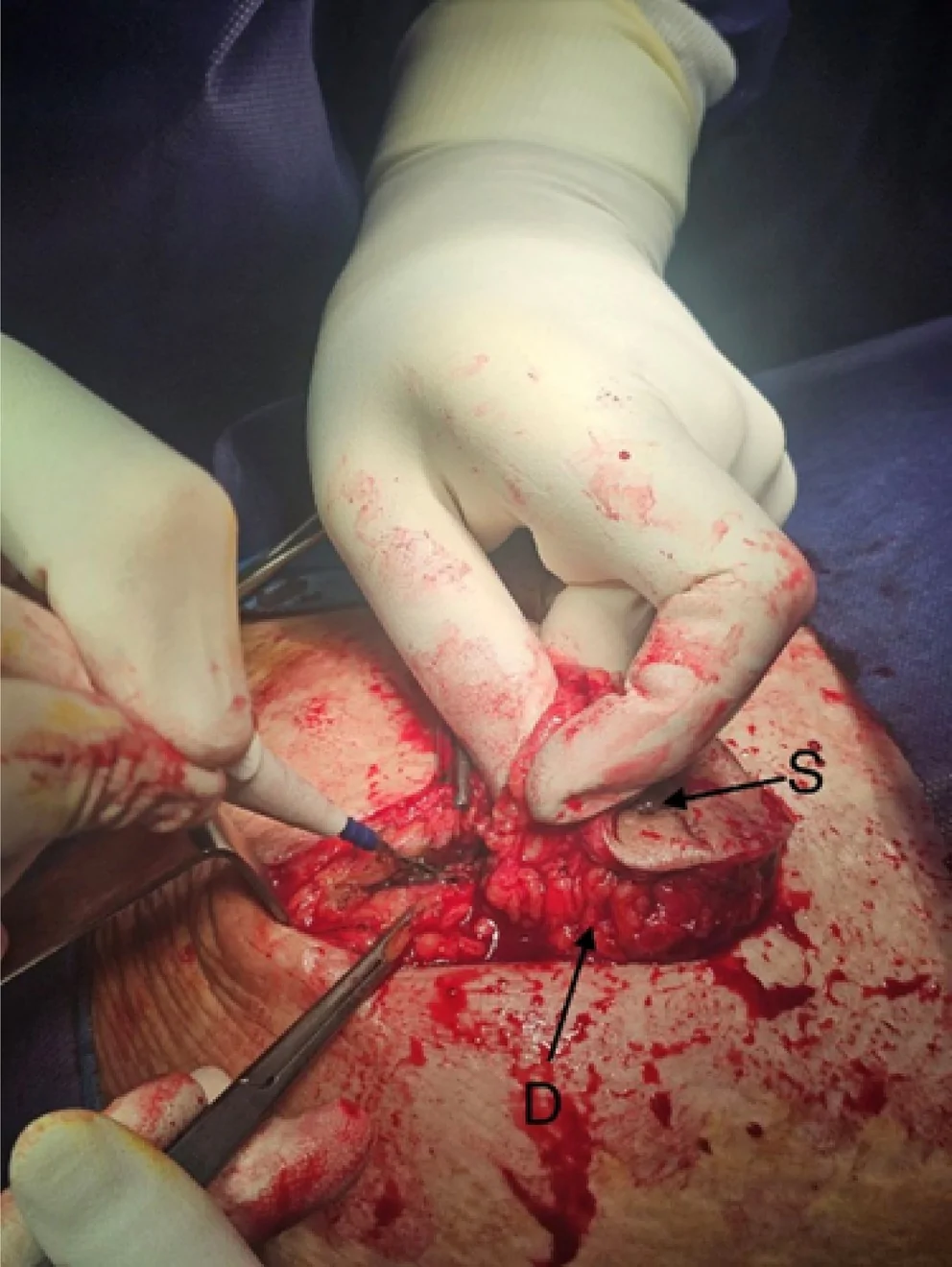
Intraoperative view of the umbilical lesion demonstrating deeper extension than initially appreciated on physical examination. The mass measured approximately 6.5 × 5 × 3.1 cm and was adherent to the anterior rectus sheath. The figure highlights the distinction between the superficial palpable component (S) and the deeper intraoperative extent (D).

The lesion, together with a rim of healthy surrounding tissue and the involved anterior rectus fascia, was completely excised (Figure 3). A total omphalectomy was performed due to the size of the lesion and fascial involvement. The fascial defect was repaired with absorbable sutures, and the abdominal wall was reconstructed to achieve an acceptable cosmetic contour. No peritoneal involvement or true hernia sac was identified intraoperatively.

**FIGURE 3:**
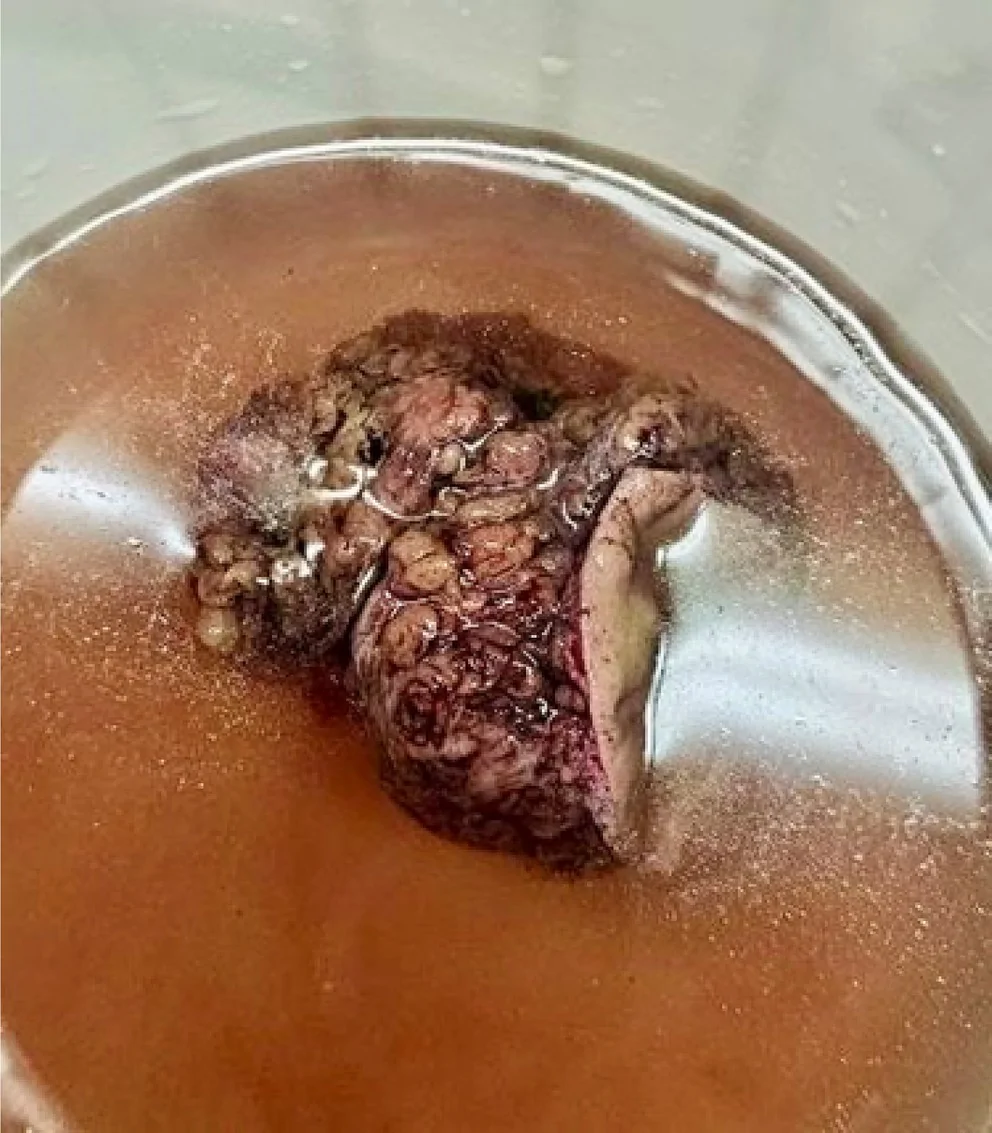
Post‐excision specimen of the umbilical lesion after complete surgical removal, including a rim of surrounding fascia. Scale bar represents 1 cm. Complete excision was performed to ensure clear margins and reduce recurrence risk.

Histological examination of the excised tissue revealed irregular glandular structures embedded within a highly cellular and vascular stroma, morphologically consistent with functional endometrial tissue (Figure 4). These findings were characteristic of endometriosis. Immunohistochemical staining showed strong positivity for CD10 and estrogen receptor (ER), confirming the diagnosis of “umbilical endometriosis”; considering her history of two cesarean sections and the clinical presentation, this lesion was classified as secondary umbilical endometriosis.

**FIGURE 4:**
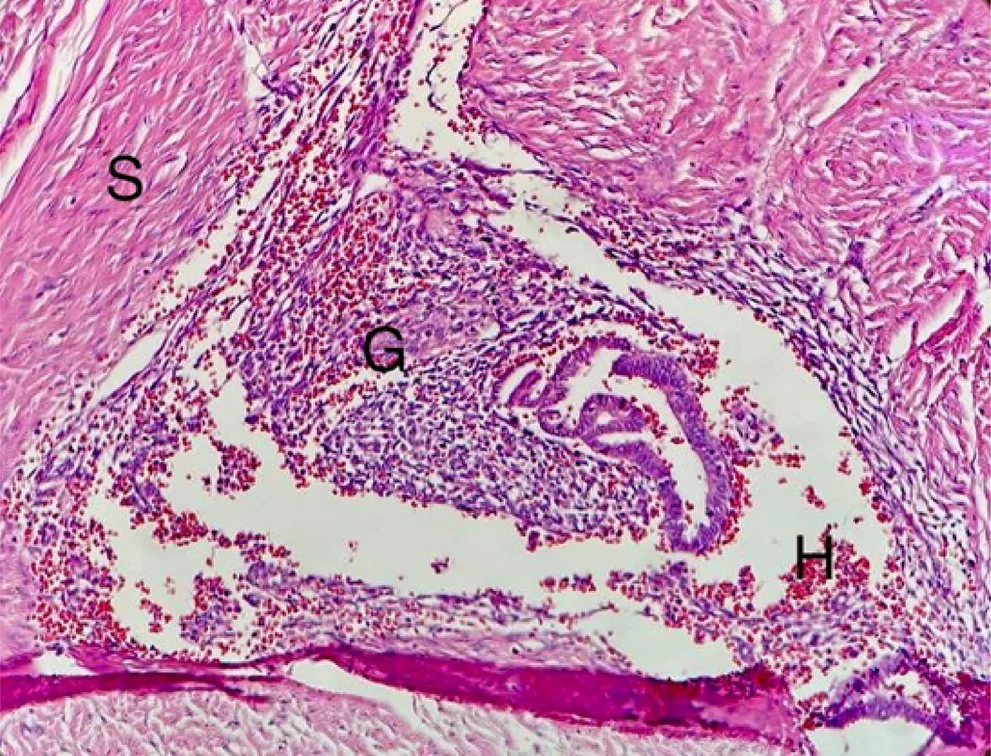
Histopathological examination showing endometrial glands (G) and stroma (S) with hemorrhage (H). Hematoxylin and eosin (H&E) stain; magnification ×40. These features are characteristic of endometriosis.

## 4 Outcome and Follow‐Up

The postoperative course was uneventful. At three‐month follow‐up, the patient reported complete resolution of her cyclical umbilical pain and bloody discharge, with high satisfaction regarding both the cosmetic outcome and her overall quality of life. No clinical evidence of recurrence was observed during this period. As a post‐operative adjunctive measure, the patient was started on oral contraceptives, although the role of hormonal therapy in preventing recurrence remains a subject of debate.

## 5 Discussion

Umbilical endometriosis (UE), also referred to as Villar's nodule, was first described by Villar in 1886 and represents a rare form of extrapelvic endometriosis characterized by the presence of endometrial glands and stroma within the umbilical region [9]. It may occur as a primary lesion in the absence of prior surgery or as secondary UE following abdominal procedures such as cesarean section, laparoscopy, or laparotomy [1, 2, 10]. Recent case reports continue to emphasize the rarity of this condition and its diagnostic challenges, particularly due to its ability to mimic other umbilical pathologies [4].

Secondary UE accounts for approximately 0.5%–1% of extragenital endometriosis cases and is strongly associated with prior abdominal surgery, supporting the theory of iatrogenic implantation of endometrial tissue [3]. In the present case, the patient had a history of two cesarean sections, which strongly supports a secondary origin. Although the patient's symptoms developed several years after her cesarean sections, delayed clinical presentation has been reported in previous studies and does not preclude classification as secondary umbilical endometriosis. UE typically affects women of reproductive age as an estrogen‐dependent condition, with a reported mean age of onset of 37.7 ± 0.98 years [6]. The patient in this report was a 43‐year‐old premenopausal woman, consistent with the expected demographic profile.

Clinically, UE most commonly presents as an umbilical mass associated with cyclical pain, swelling, and sometimes bleeding or discharge [2]. Umbilical swelling is reported in up to 90% of cases, cyclical pain in 82%, and bleeding or discharge in nearly half [2, 11]. In this case, the combination of cyclical periumbilical pain and bloody discharge represented a typical but initially unrecognized presentation, contributing to diagnostic delay.

A major diagnostic challenge in this case was the preoperative misinterpretation as an irreducible umbilical hernia. Ultrasound suggested a fascial defect with protruding mesenteric fat, leading to a provisional diagnosis of incarcerated hernia. The sonographic appearance was likely influenced by surrounding fibrosis and chronic inflammatory changes, which may have simulated protruding preperitoneal fat through a fascial defect. In retrospect, the presence of two sinus‐like openings and cyclic bloody discharge should have broadened the differential diagnosis beyond an incarcerated umbilical hernia. However, intraoperative findings revealed a firm fibrotic lesion extending into the anterior rectus fascia without a true hernia sac. This highlights the limited specificity of imaging in UE. Although ultrasound is useful for assessing lesion size and depth, it cannot reliably establish the diagnosis. Magnetic resonance imaging (MRI) provides superior soft‐tissue characterization and may improve preoperative diagnostic accuracy in selected patients with equivocal clinical or ultrasonographic findings [5, 6, 12]. Therefore, UE should always be considered in the differential diagnosis of umbilical masses in women of reproductive age, particularly when symptoms are cyclical. Although fine‐needle aspiration cytology has been described as an adjunctive diagnostic tool, it was not performed in the present case because surgical exploration was already indicated based on the presumed diagnosis of an irreducible umbilical hernia.

Another important feature of this case was the unusually large lesion size. Most reported UE nodules measure between 0.5 and 3 cm [7]. In contrast, the lesion in this patient measured approximately 6.5 × 5 × 3.1 cm and demonstrated extension into the anterior rectus fascia. This discrepancy between clinical and intraoperative findings suggests that deeper fascial involvement may lead to underestimation of lesion size preoperatively and increases surgical complexity.

Histopathological examination remains the gold standard for diagnosis [5, 6]. Typical findings include endometrial glands within a cellular and vascular stroma resembling functional endometrium. Immunohistochemical staining for CD10 and estrogen receptors further supports the diagnosis [8, 13]. In this case, both histopathological and immunohistochemical findings confirmed UE.

Surgical excision is the definitive treatment for UE, providing both diagnostic confirmation and symptom resolution while minimizing recurrence risk [7, 8]. Although conservative umbilicus‐preserving procedures have been described, total omphalectomy with en bloc excision of the involved fascia was performed in this patient due to the lesion's large size and fascial involvement. This approach ensured complete resection and reduced the likelihood of residual disease. Primary fascial closure without mesh was feasible because complete tension‐free approximation was achieved following excision of the involved fascia.

Comparison with previously published reports highlights the uniqueness of this case. The combination of a large lesion size, deep fascial involvement, and preoperative misdiagnosis as an irreducible hernia is rarely reported together. This underscores the importance of maintaining a high index of suspicion for UE in atypical umbilical masses, particularly in patients with prior abdominal surgery.

Concurrent pelvic endometriosis has been reported in approximately 13%–15% of UE cases [6, 14]. However, in the absence of pelvic symptoms, routine additional imaging is not universally recommended. In this case, the patient had no clinical features suggestive of pelvic endometriosis, and no further evaluation was performed. The patient was advised to undergo routine gynecological follow‐up, and further evaluation for pelvic endometriosis would be considered if pelvic symptoms developed.

Postoperatively, the patient was started on oral contraceptive therapy by the treating gynecologist, primarily with the aim of reducing the risk of recurrence following surgical excision. However, current evidence remains insufficient to support routine postoperative hormonal therapy for all patients after complete surgical excision, and the decision to initiate hormonal suppression should be individualized based on patient characteristics, symptoms, and clinical circumstances. In selected patients, careful clinical follow‐up without hormonal therapy may also be an appropriate management strategy. In this case, although postoperative hormonal therapy was administered, the patient continued to experience regular menstrual cycles during follow‐up; therefore, the absence of recurrent cyclical symptoms cannot be attributed solely to hormonal suppression. Furthermore, hormonal therapy may theoretically delay the recognition of recurrent cyclical symptoms, emphasizing the importance of long‐term clinical surveillance regardless of whether hormonal treatment is used. Nevertheless, the potential contribution of postoperative hormonal therapy to symptom control should be considered when interpreting short‐term postoperative outcomes.

The recurrence rate of UE after surgical excision has been reported to range from 5.4% to 27% [7, 8]. Although no recurrence was observed during the three‐month follow‐up period, longer follow‐up is necessary to assess long‐term durability. The patient reported complete resolution of symptoms and high satisfaction with both the cosmetic outcome and overall quality of life, although no standardized patient‐reported cosmetic outcome measure was used. The patient reported complete resolution of symptoms and high satisfaction with both the cosmetic outcome and quality of life. Long‐term clinical follow‐up was planned, and the patient was counseled regarding the possibility of recurrence and advised to seek medical attention if new cyclical symptoms or umbilical swelling developed.

This case has several limitations. Additional evaluation for concurrent pelvic or extra‐umbilical endometriosis was not performed; therefore, asymptomatic disease cannot be excluded. The follow‐up period was limited to 3 months, which is insufficient to assess long‐term recurrence. In addition, postoperative hormonal therapy may have influenced symptom control and should be considered when interpreting early outcomes. Future studies with longer follow‐up periods and standardized patient‐reported cosmetic outcome measures are needed to better evaluate long‐term outcomes after surgical management of secondary umbilical endometriosis.

## 6 Conclusion

Umbilical endometriosis is an uncommon but important differential diagnosis of umbilical masses in women of reproductive age, particularly in those with prior abdominal surgery. Cyclical symptoms should raise clinical suspicion. Complete surgical excision with histopathological confirmation provides definitive diagnosis and effective treatment, especially in large lesions with fascial involvement.

## Author Contributions

**Babak Mamaghani:** investigation, resources, supervision, writing – original draft. **Mohsen Hasanzadeh:** investigation, resources, supervision, writing – original draft. **Amirmohammad Khodaei:** investigation, project administration, software, writing – review and editing. **Zeinab Shakibaee Fard:** investigation, software, supervision, writing – review and editing.

## Funding

The authors have nothing to report.

## Ethics Statement

The authors have nothing to report. The patient provided consent for the writing and publication of this article.

## Consent

Apart from age and gender, no identifying details were included in the manuscript. Written informed consent was obtained from the patient for the publication of this case report and any accompanying images, in accordance with the journal's patient consent policy.

## Conflicts of Interest

The authors declare no conflicts of interest.

## Data Availability

The data that support the findings of this study are available on request from the corresponding author. The data are not publicly available due to privacy or ethical restrictions.
